# Supplementary material for: ASH1L histone methyltransferase regulates the handoff between damage recognition factors in global-genome nucleotide excision repair
Source: Nat Commun. 2017 Nov 6;8:1333. doi: 10.1038/s41467-017-01080-8 (PMC5673894; doi:10.1038/s41467-017-01080-8)
Supplement: Supplementary file 1 — Supplementary Information [file 41467_2017_1080_MOESM1_ESM.pdf]

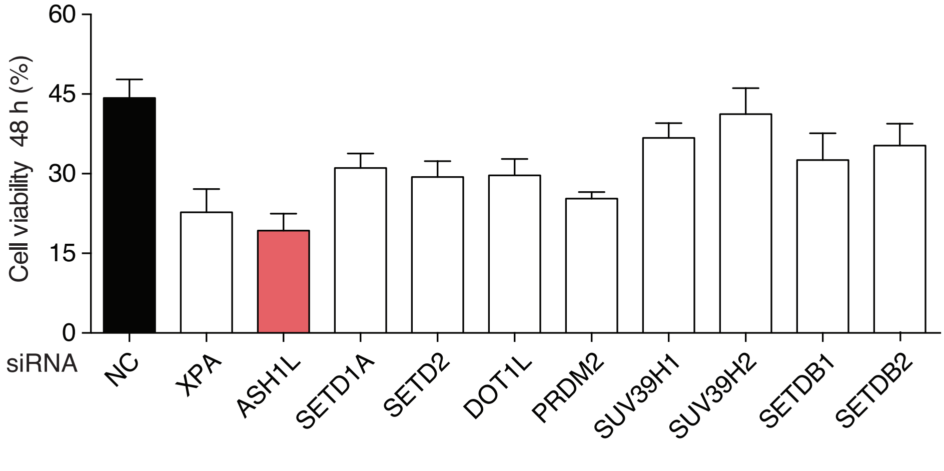

Supplementary Figure 1. Effect of histone methyltransferase depletion on UV sensitivity HeLa cells were transfected with siRNA targeting the following transcripts: XPA, ASH1L, SETD1A, SETD2, DOT1L, PRDM2, SUV39H1, SUV39H2, SETDB1 and SETDB2. Control cells were transfected with non-coding RNA (NC). After an incubation of 48 h, cells were irradiated with UV-C (5 J m^{-2}) and their survival was assessed by the Alamar Blue assay following another 48 h. All values are expressed as the percentage of cell viability detected with corresponding unirradiated controls (n = 3, each independent experiment with 4 replicates).

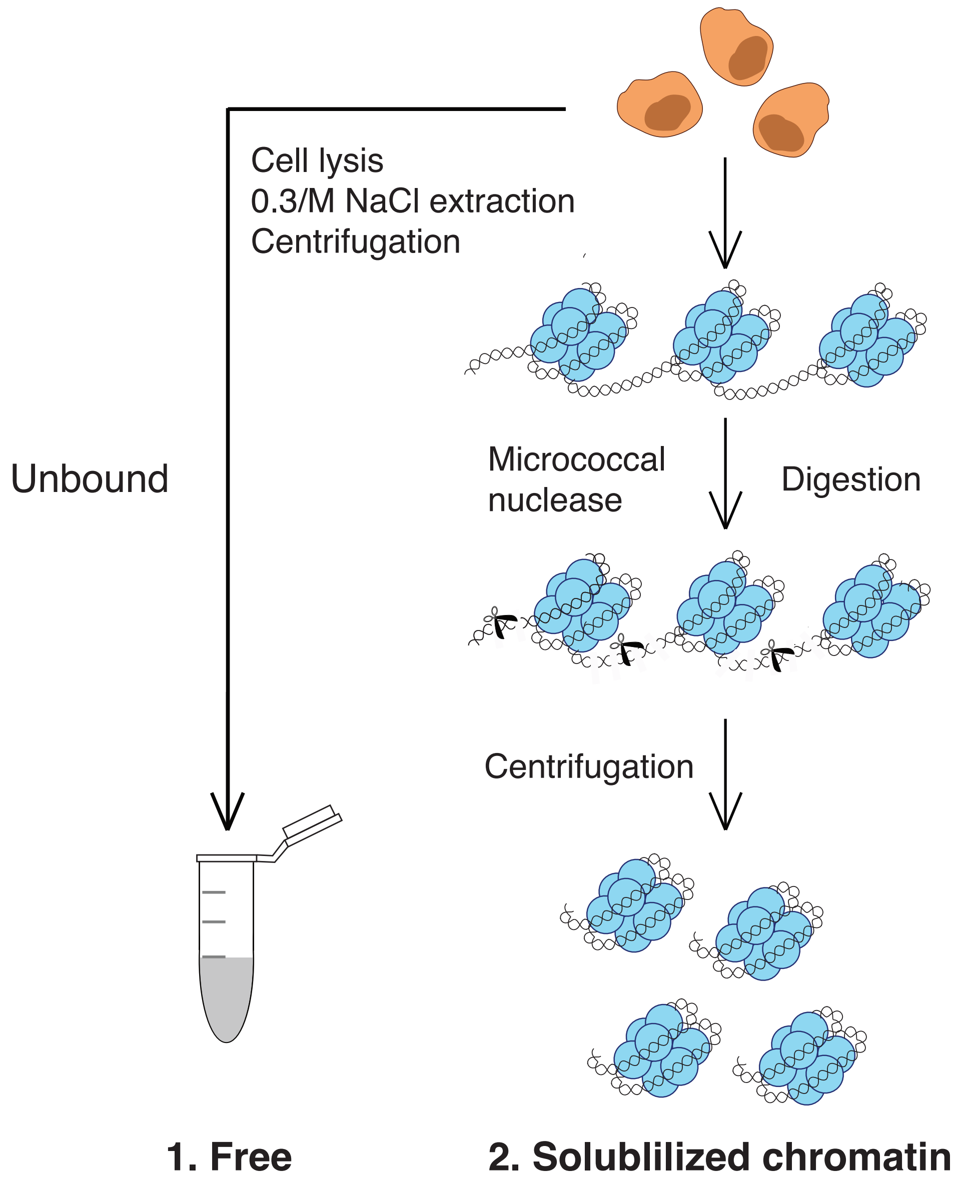

Supplementary Figure 2. Solubilization of chromatin by nuclease digestion

Flow diagram illustrating the chromatin analyses. HeLa cells were lysed and extracted with buffer containing 0.3 M NaCl to remove in the supernatant free proteins that are not associated with chromatin or only loosely bound to chromatin. The remaining chromatin pellet was solubilized by incubation with a saturating MNase concentration. By cleaving internucleosomal linker regions, this nuclease treatment generates solubilized chromatin containing mononucleosomes that are amenable to immunoprecipitation or other biochemical analyses. For chromatin immunoprecipitation (ChIP) assays, the cells were fixed by the addition of 1% (vol/vol) formaldehyde before extraction and solubilization.

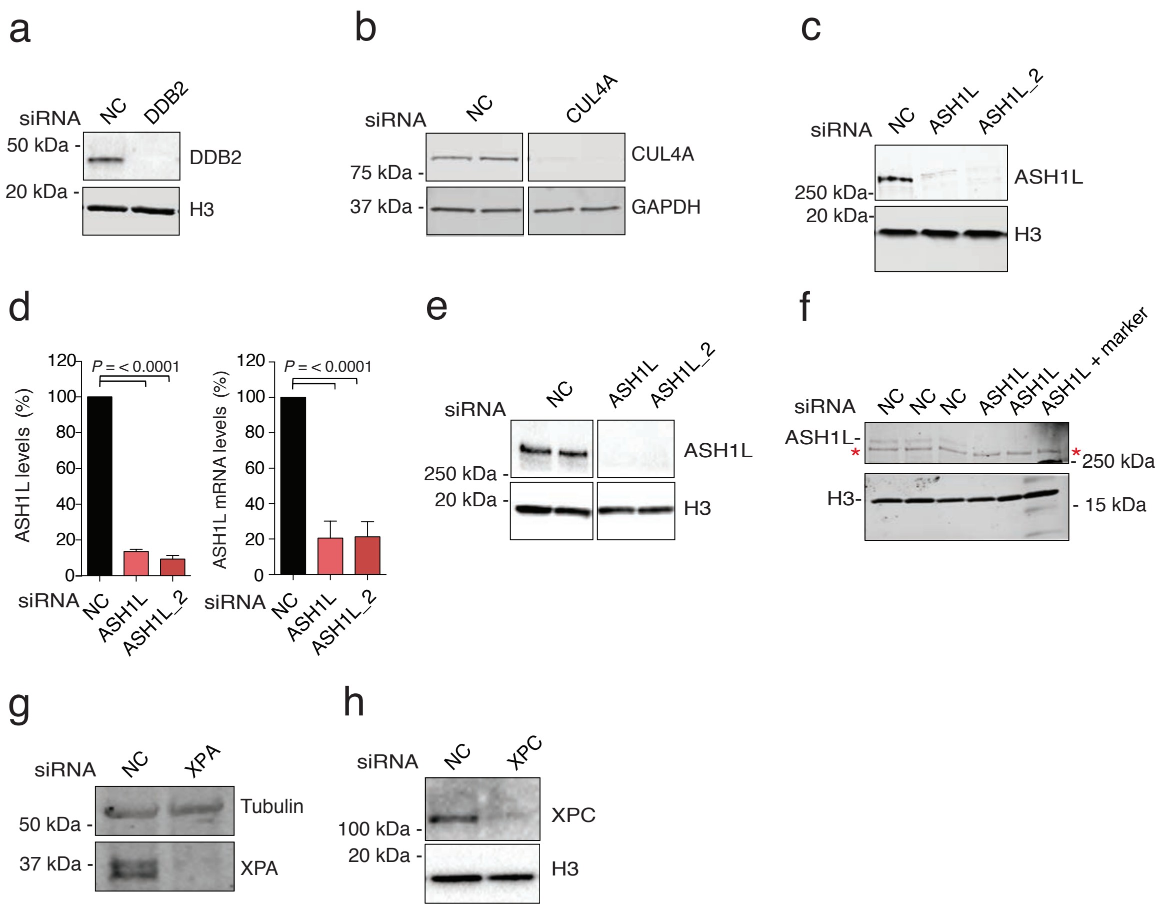

Supplementary Figure 3. Efficiency of protein depletion 48 h after transfection with siRNA

(a) Down regulation of DDB2 protein in HeLa cells. Immunoblot of whole-cell lysates with histone H3 as the loading control. siNC, non-coding control RNA.

(b) Down regulation of CUL4A in HeLa cells. Immunoblot of whole-cell lysates in duplicate with GAPDH as the loading control.

(c) ASH1L down regulation in HeLa cells using two different siRNA sequences. Immunoblot of whole-cell lysates with H3 as the loading control.

(d) The level of ASH1L protein and corresponding mRNA transcript was quantified over three independent experiments (error bars show s.e.m.).

(e) ASH1L down regulation in U2OS cells. Immunblot of whole cell lysate with histone H3 as the loading control.

(f) Non-specific band, indicated by the asterisks, appearing when immunoblotting the chromatin fraction against ASH1L. The chromatin of HeLa cells transfected with siASH1L

(or non-coding siNC) was immunoblotted to demonstrate that only the upper of two closely migrating bands represents the ASH1L protein.

(g) XPA down regulation in HeLa cells, tubulin was used as the loading control.

(h) XPC down regulation in HeLa cells, H3 was used as the loading control.

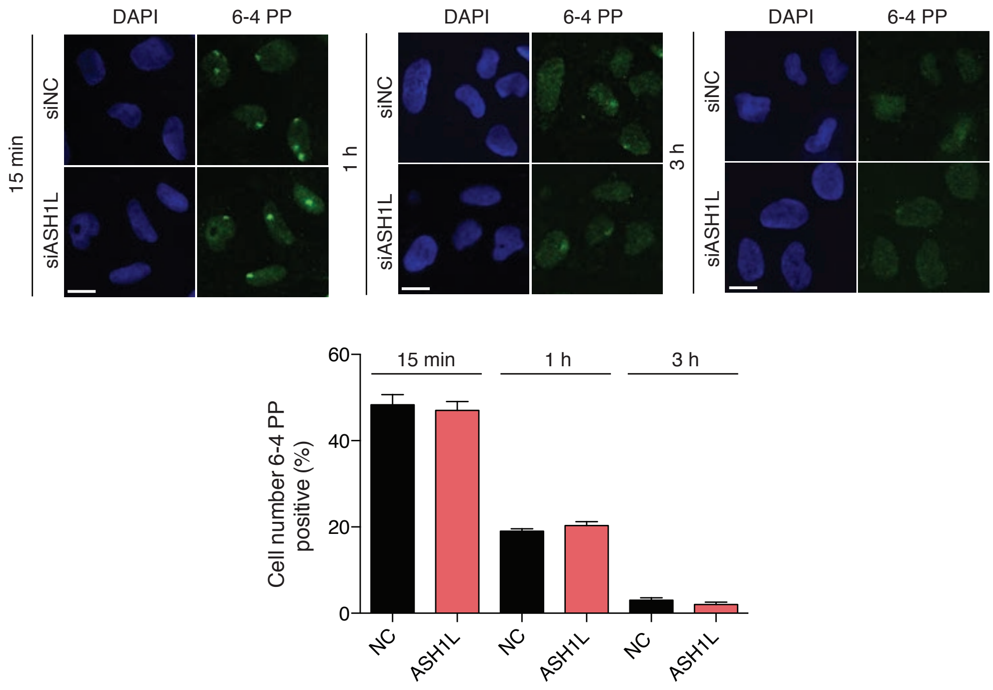

Supplementary Figure 4. Time course of 6-4PP repair in UV lesion spots

Immunofluorescence detection of 6-4PPs in U2OS at different times after UV radiation (100 J m^{-2}) through 5-μm filter pores. DNA was stained with DAPI. This time course experiment shows that most 6-4PPs are removed from the UV lesion spots within 2-3 h after UV irradiation. Scale bar = 15 μm.

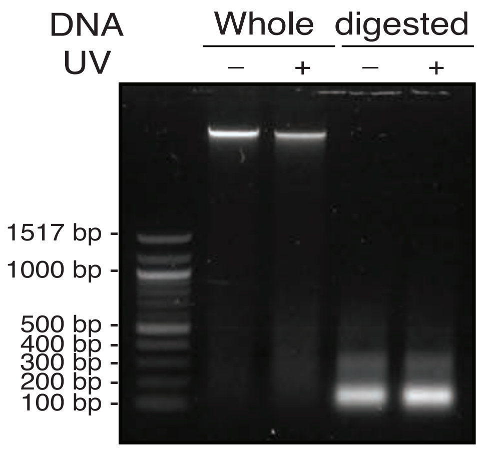

Supplementary Figure 5. Mononucleosomes for immunoprecipitation studies

Ethidium bromide staining of an agarose gel demonstrating the complete digestion of genomic DNA, to generate isolated nucleosome core particles, with a saturating MNase concentration. By this enzymatic digestion, the whole chromatin is converted to nucleosome core fragments of 147 base pairs.

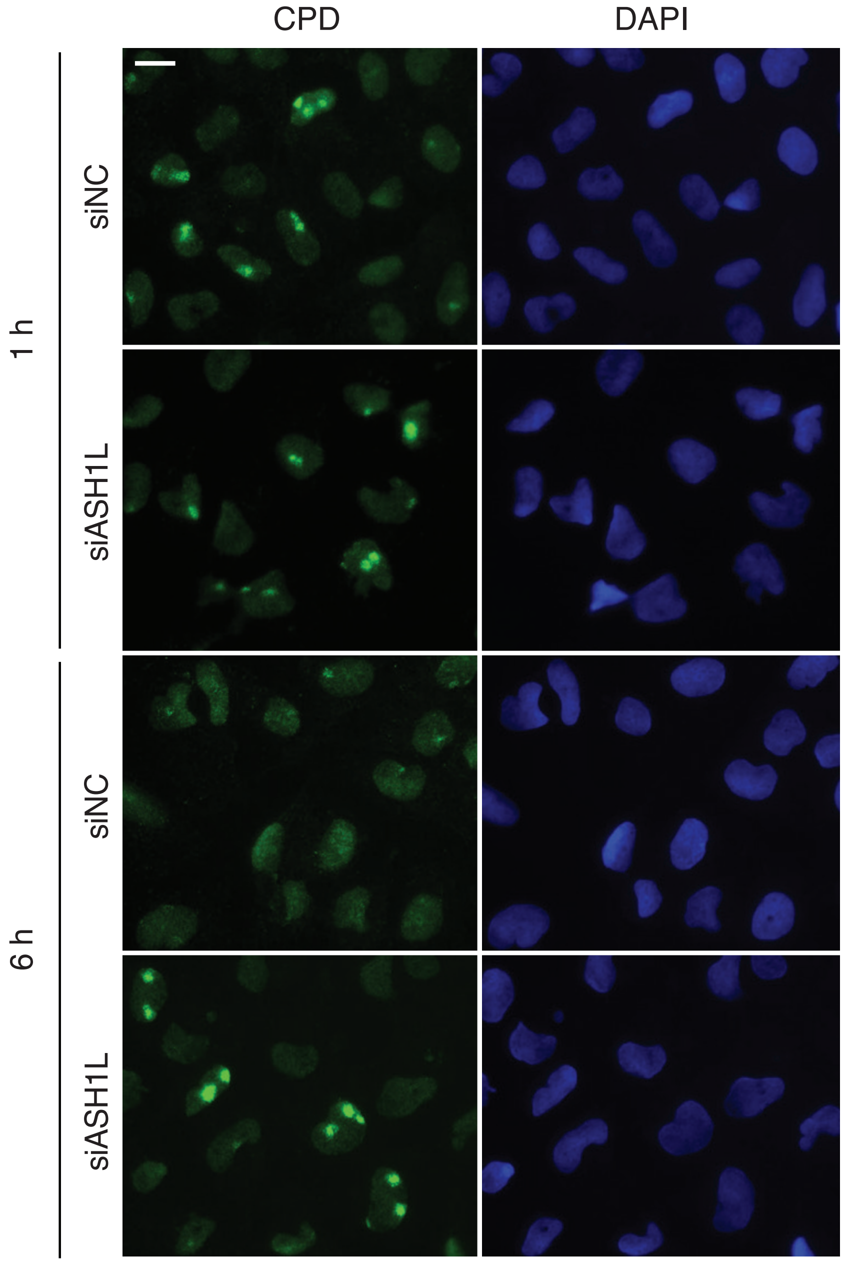

Supplementary Figure 6. Wide-field views of immunofluorescence detection of CPDs
U2OS cells were transfected with siASH1L or siNC 48 h before UV radiation (100 J m^{-2}) through 5-μm filter pores. CPDs were stained with antibodies and DNA with DAPI. The figure shows representative images of cells after repair incubations of 1 and 6 h. At 6 h of repair, CPDs are barely detectable in control cells (transfected with siNC) but still appear as persistent bright spots in ASH1L-depleted cells. Scale bar = 15 μm.

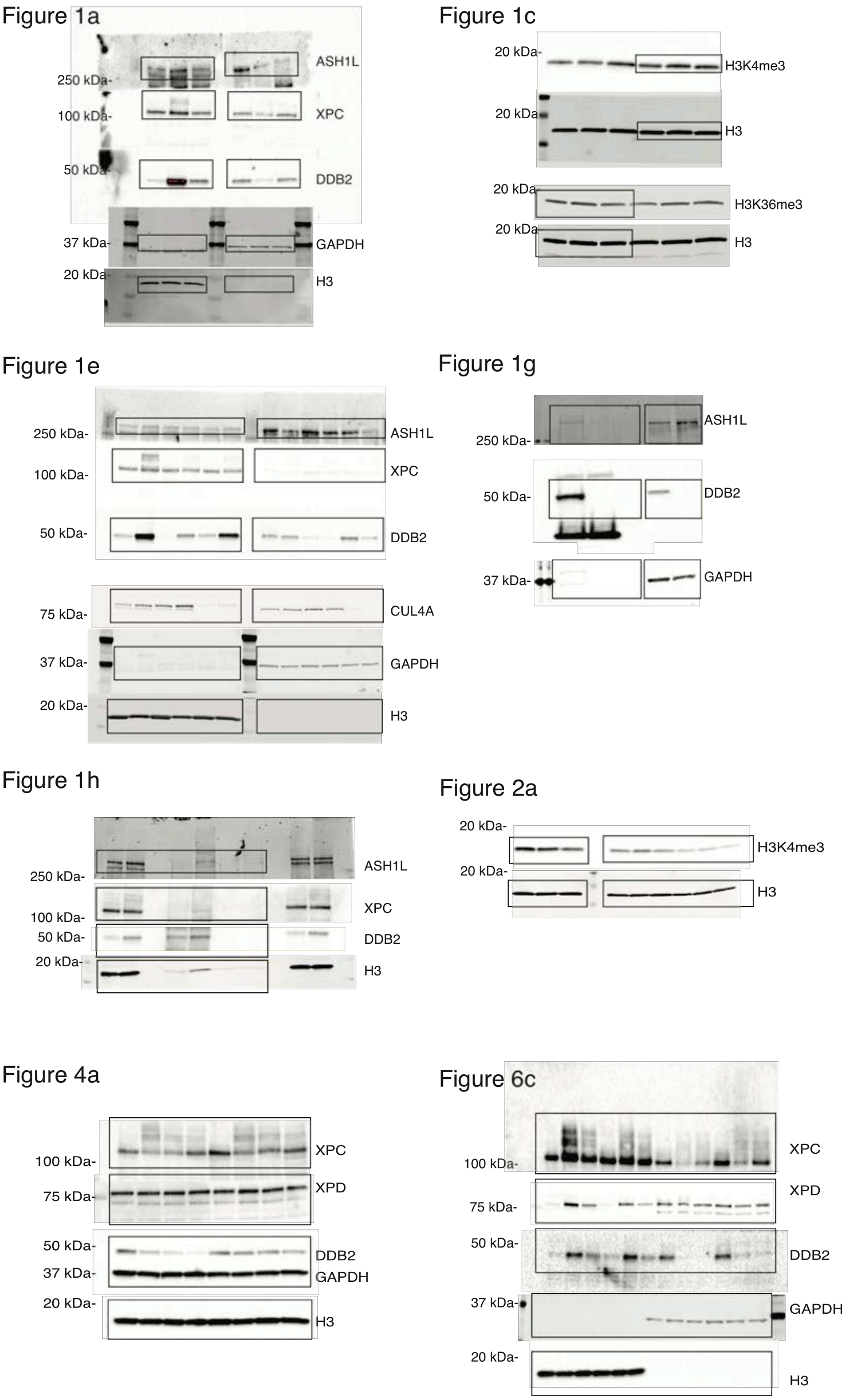

Supplementary Figure 7. Full blots of Fig. 1, 2, 4 and 6 as indicated

Rectangles show the portion of each blot used for the respective figures.

Figure 7a

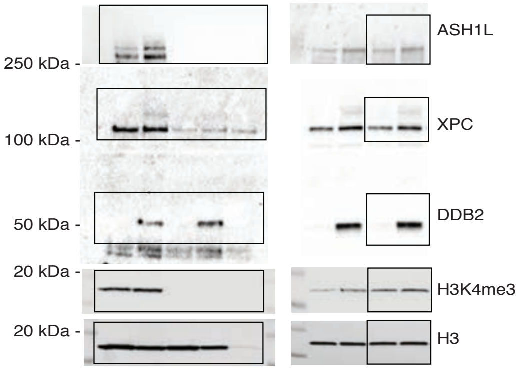

Figure 7c

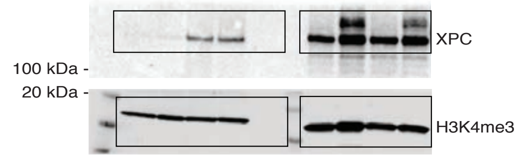

Figure 7e

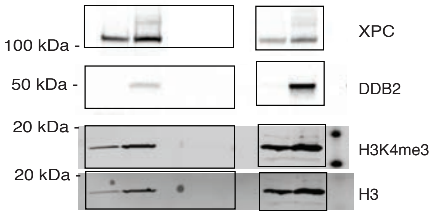

Figure 7f

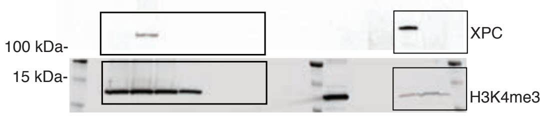

Figure 8b

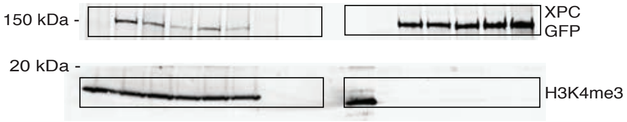

Figure 8e

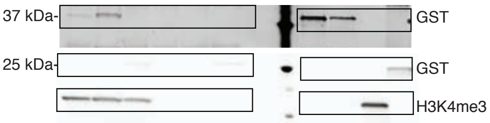

Supplementary Figure 8. Full blots of Fig. 7 and Fig. 8 as indicated

Rectangles show the portion of each blot used for the respective figures.

Supplementary Figure 3a

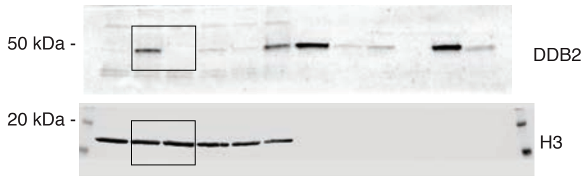

Supplementary Figure 3b

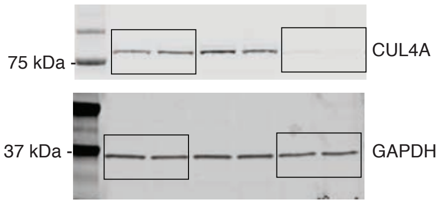

Supplementary Figure 3c

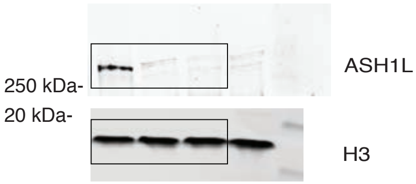

Supplementary Figure 3e

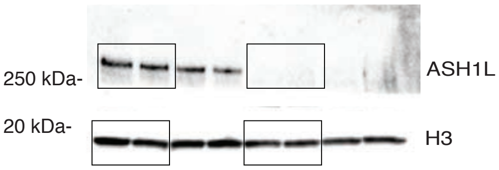

Supplementary Figure 3f

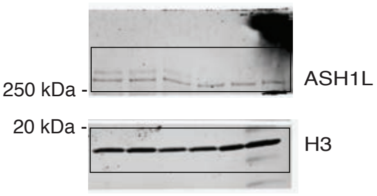

Supplementary Figure 3g

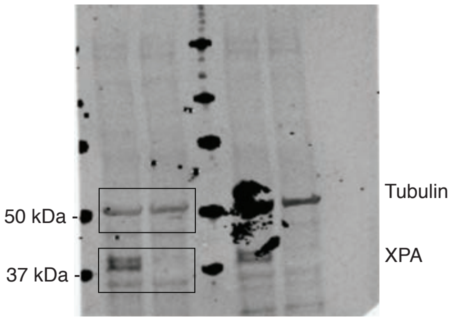

Supplementary Figure 3h

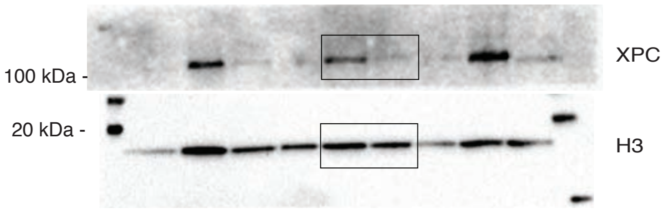

Supplementary Figure 9. Full blots of Supplementary Fig. 3 as indicated

Rectangles show the portion of each blot used for the respective figures.

Supplementary Table 1. Sequences of siRNA reagents

siRNA	Sequence	Source, identifier
Non-coding (NC)	5’-AAUUCUCCGAACGUGUCACGU-3’	Qiagen, SI03650325
DDB2	5’-GGAUCAAGCAGUUAUUUGATT-3’	Qiagen, SI02664823
XPC	5’-GCAAAUGGCUUCUAUCGAATT-3’	Qiagen, SI00066227
ASH1L	5’-TCGACTGTTATCTCACATTAA-3’	Qiagen, SI00122094
ASH1L_2	5’-CAGGCTGTCGTATCAATGCAA-3’	Qiagen, SI00122087
SETD2	5’-GAAACCGUCUCCAGUCUGUTT-3’	Microsynth, 2463700
PRDM2	5’-AACAGACCGGUUCAAUAUAAATT-3’	Microsynth, 2055934
CUL4A	5’- TTCGAAGGACATCATGGTTCA-3’	Microsynth, 2759182
SETB1	5’-CAGCATGCGAATTCTGGGCAA-3’	Qiagen, SI05062666
SETB2	5’-TACCAGGTTTAGAATGGTATA-3’	Qiagen, SI00141386
SUV39H1	5’-CAGGTGTACAACGTCTTCATA-3’	Qiagen, SI02665019
SUV39H2	5’-AAGCGTTAAGCTGATAATGTA-3’	Qiagen, SI02665026
SETD1A	5’-CAGCGTATTATGAAAGCTGGA	Qiagen, SI00453180
DOT1L	5’-CCGGCCTTCGTCGAAGCAGAA-3’	Qiagen, SI00143311
XPA	5’-GCUACUGGAGGCAUGGCUATT-3’	Microsynth, 2343554

Supplementary Table 2. Sequences of quantitative RT-PCR primers

Target	Sequence	Source
GAPDH	fw-AGCCACATCGCTCAGACAC	Microsynth
	rev-GCCCAATACGACCAAATCC	Microsynth
B2M	fw-ATGTCTCGCTCCGTGGCCTTA	Microsynth
	rev-ATCTTGGGCTGTGACAAAGTC	Microsynth
ASH1L	fw-TATTGAAGTGGCGGGCAG	Microsynth
	rev-CACACAGTCACCCTGACGAA	Microsynth

Supplementary Table 3. List of antibodies

Antibody	Source	Catalog number	Dilutions
Mouse anti-DDB2	Abcam	Cat#ab51017	WB, 1:200; IF, 1:50; ChIP, 1:20
Rabbit anti-H3K4me3	Abcam	Cat#ab8580	WB, 1:1,000; IF, 1:500; IP, 1:50
Rabbit anti-H3k36me3	Abcam	Cat#ab9050	WB, 1:1,000
Rabbit anti-SETD2	Abcam	Cat#ab31358	WB, 1:500
Rabbit anti-GFP	Abcam	Cat#ab290	WB, 1:1,000
Mouse anti-XPC	Abcam	Cat#ab6264	WB, 1:1,000
Mouse anti-GAPDH	Abcam	Cat#ab9484	WB, 1:1,000
Mouse anti-alfa-tubulin	SIGMA-ADRICH	Cat#T5168	WB, 1:1,000
Mouse anti-CPD	Cosmo Bio Co LTD	Cat#NMDND001	IF, 1:1,000; ELISA 1:5,000
Mouse anti-6-4PP	Cosmo Bio Co LTD	Cat#NMDND002	IF, 1:500; ELISA, 1:2,000
Rabbit anti-ASH1L	Santa Cruz	Cat#sc-98301	WB, 1:1,000
Goat anti-H3	Santa Cruz	Cat#sc-8654	WB, 1:1,000; IP, 1:20
Rabbit anti-XPC	SIGMA-ADRICH	Cat#X1129	IF, 1:100; IP, 1:20
Rabbit anti-GST	SIGMA-ADRICH	Cat#G7781	WB, 1:2,000
Rabbit anti-XPD	GeneTex	Cat#GTX108948	WB, 1:1,000; IF, 1:100
Rabbit Anti-CLU4A	Thermo Scientific	Cat#PA5-17101	WB, 1:1,000
Alexa Fluor 488 anti-rabbit	Thermo Scientific	Cat#A-32731	IF, 1:400
Alexa Fluor 488 anti-mouse	Thermo Scientific	Cat#A-11001	IF, 1:400
Alexa Fluor 594 anti-rabbit	Thermo Scientific	Cat#A-11037	IF, 1:400
Alexa Fluor 594 anti-mouse	Thermo Scientific	Cat#A-11005	IF, 1:400
F(ab')2 secondary antibody, biotin	Thermo Scientific	Cat#31803	ELISA, 1:2,000
Anti-Mouse IgG (Fab specific)	SIGMA-ADRICH	Cat#A2304	WB, 1:10,000
Anti-Rabbit IgG (whole molecule)	SIGMA-ADRICH	Cat#A0545	WB, 1:10,000
IRDye 800CW goat anti-mouse IgG	LI-COR	Cat#925-32210	WB, 1:10,000
IRDye 800CW goat anti-rabbit IgG	LI-COR	Cat#925-32211	WB, 1:10,000
IRDye 800CW donkey anti-goat IgG	LI-COR	Cat#925-32214	WB, 1:10,000
IRDye 680LT goat anti-mouse IgG	LI-COR	Cat#925-68020	WB, 1:10,000
IRDye 680LT goat anti-rabbit IgG	LI-COR	Cat#925-68021	WB, 1:10,000

WB, Western blots; IF, immunofluorescence; ChIP, chromatin immunoprecipitation; IP, immunoprecipitation.
